# Large-scale gene discovery in the pea aphid *Acyrthosiphon pisum *(Hemiptera)

**DOI:** 10.1186/gb-2006-7-3-r21

**Published:** 2006-03-10

**Authors:** Beatriz Sabater-Muñoz, Fabrice Legeai, Claude Rispe, Joël Bonhomme, Peter Dearden, Carole Dossat, Aymeric Duclert, Jean-Pierre Gauthier, Danièle Giblot Ducray, Wayne Hunter, Phat Dang, Srini Kambhampati, David Martinez-Torres, Teresa Cortes, Andrès Moya, Atsushi Nakabachi, Cathy Philippe, Nathalie Prunier-Leterme, Yvan Rahbé, Jean-Christophe Simon, David L Stern, Patrick Wincker, Denis Tagu

**Affiliations:** 1 INRA Rennes, UMR INRA-Agrocampus BiO3P, BP 35327, F-35653 Le Rheu Cedex, France; 2 INRA, URGI - Genoplante Info, Infobiogen, 523 place des Terrasses, F-91000 Evry, France; 3 Biochemistry Department, University of Otago, PO Box 56, Dunedin, New Zealand; 4 GENOSCOPE and CNRS UMR 8030, Centre National de Séquençage, 2 rue Gaston Crémieux, F-91000 Evry Cedex, France; 5 USDA, Agricultural Research Service, US Horticultural Research Laboratory, 2001 South Rock Road, Fort Pierce, FL 34945, USA; 6 Department of Entomology, Kansas State University, Manhattan, KS 66506, USA; 7 Institut Cavanilles de Biodiversitat i Biologia Evolutiva (ICBIBE), Universitat de Valencia, Apartado de Correos 2085, 46071 Valencia, Spain; 8 Environmental Molecular Biology Laboratory, RIKEN, 2-1 Hirosawa, Wako, Saitama 351-0198 Japan; 9 INRA Lyon, UMR INRA-INSA BF2I, INSA Bâtiment Louis-Pasteur, 20 avenue A. Einstein, 69621 Villeurbanne cedex, France; 10 Department of Ecology and Evolutionary Biology, Princeton University, Princeton, NJ 08544, USA; 11 Current address: Instituto Valenciano de Investigaciones Agrarias (IVIA), Proteccion Vegetal y Biotecnologia, Lab Entomologia, 46113 Moncada, Valencia, Spain

## Abstract

Aphids are the leading pests in agricultural crops. A large-scale sequencing of 40,904 ESTs from the pea aphid *Acyrthosiphon pisum *was carried out to define a catalog of 12,082 unique transcripts. A strong AT bias was found, indicating a compositional shift between *Drosophila melanogaster *and *A. pisum*. An *in silico *profiling analysis characterized 135 transcripts specific to pea-aphid tissues (relating to bacteriocytes and parthenogenetic embryos). This project is the first to address the genetics of the Hemiptera and of a hemimetabolous insect.

## Abstract 2

A large-scale sequencing analysis of the Hemiptera *Acyrthosiphon pisum*expressed sequence tags corresponding to about 12,000 unique transcripts is described, along with an *in silico *profiling analysis that identifies 135 aphid tissue-specific transcripts.

## Background

Many of the 4,500 aphid species (Hemiptera: Aphididae) cause serious physical and economic damage to cultivated and ornamental plants throughout the world. Aphids affect plant growth not only directly through feeding on phloem sap but also as vectors of plant viruses [1]. The extent of losses due to aphids is difficult to evaluate as it depends on multiple factors such as aphid species or virus isolate, crop, location, and year. On many crops insecticides provide a simple solution for aphid control. The large-scale application of such chemicals is becoming increasingly unacceptable, however, and their use needs to be optimized in an environmentally acceptable way so as to maintain both farm incomes and an adequate food supply. This is even more important in face of the increasing number of aphid species (more than 20) that have developed resistant populations against most insecticides [2]. The use of plant varieties resistant to aphids is an alternative to chemical control. But again, aphids have developed biotypes able to overcome the few sources of aphid resistance in plants [3]. It is therefore necessary to develop new targets for specific and effective molecules against aphids and to assess their sustainability through a careful analysis of the adaptive potential of these insects.

The harmful effects of aphids depend on four main traits: first, a high intrinsic rate of increase driven largely by parthenogenesis and telescoping of generations [4]; second, the capacity to adapt physiologically to variable phloem sap content between host plants [5], which is partly conferred by bacterial endosymbionts; third, the facultative production of winged dispersal forms [6], which allows the rapid colonization of new environments; and fourth, the vectoring of many plant viral pathogens [7,8].

**Figure 1:**
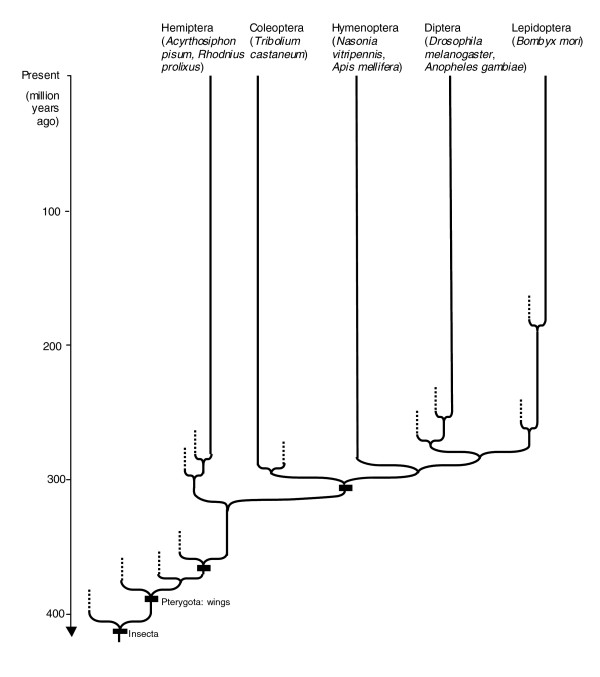
Schematic phylogenetic tree representing the insect Orders comprising species where genome sequencing projects have been completed or are in an advanced stage. The figure is a greatly simplified version of a phylogeny shown in [9] representing the largely agreed relationships between these Orders, plus the major evolutionary transitions for insects (as deduced by synamorphic characters, that is, novel characters derived from preexisting ones) along a time scale expressed in millions of years from present. For each Order with species involved in a genome sequencing project, the node corresponding to its separation from its most closely related order (extant or extinct) is shown (dashed lines represent sister clades).

A basic understanding of aphid biology and applied research both require a better characterization of the physiological, cellular, and molecular mechanisms specific to these insects. Aphid sequences are poorly represented in gene databases: when beginning this study (in November 2003) only 6,491 nucleotide sequences (including a majority of anonymous molecular markers) were found in GenBank for the whole Aphididae family. Although several other insect genomes are now available, they all belong to orders that undergo complete metamorphosis (the Holometabola) and share a common ancestor about 300 million years ago (Figure 1). The evolutionary divergence of aphids (which belong to the Hemiptera and do not undergo complete metamorphosis) from the Holometabola occurred about 330 million years ago [9], so their genome is expected to differ substantially from that of other insects. Genomic data for non-holometabolous insects (aphids will be the first complete sequence in that category along with the bug *Rhodnius prolixus*) will have great value for understanding aphid biology.

The International Aphid Genomics Consortium has selected the pea aphid *Acyrthosiphon pisum *as the model aphid species (it has a genome of four holocentric chromosomes and approximately 525 Mb), and its genome sequencing project has recently been funded. We present here a collection of 40,904 high-quality annotated expressed sequence tags (ESTs) generated from different organs of the pea aphid. These ESTs form 12,082 different contigs and singletons, and represent a first significant step towards the comprehensive description of cellular functions involved in aphid biology.

## Results

### Unique transcript catalog for A. pisum

We generated 47,443 ESTs from nine cDNA libraries corresponding to six different biological sources (Table 1) representing about 28 Mb. Sequences were filtered in order to remove rRNA contaminants, short sequences, *Escherichia coli *and *Buchnera aphidicola *sequences (see Materials and methods and Table 2). From 47,443 sequences, 40,904 (86%) were retained for further analysis. Some virus sequences (213) were detected in the collection and were eliminated afterwards. Cytochrome oxidase subunits I and III transcripts encoded by the mitochondrion (289 and 119 ESTs respectively) were detected as well. The average sequence size per library varied from 363 bp (ApHL3SD) to 871 bp (ApBac).

Clusters and contigs were produced from the set of 40,904 ESTs, together with three cDNAs retrieved from GenBank. Redundancy (defined as one minus the number of ESTs forming singletons and contigs/total number of ESTs) ranged from 30% (antennae) to 86% and 92% (bacteriocytes and parthenogenetic embryos respectively) (see Table 2). A contig version (called v2) determined from the whole collection of 40,907 ESTs and cDNAs is available [10]. A total of 12,082 different assembled sequences were produced with a global redundancy of 70.5%. Despite this high redundancy, contigs composed of only one EST (singletons) were more abundant (7,782 contigs or 64%) than contigs made of more than one EST (4,300 contigs or 36%) (see Table 2). In this paper, we will call 'unique transcripts' the collection of 12,082 different assembled *A. pisum *sequences composed of singletons and contigs.

### Functional annotation

**Figure 2:**
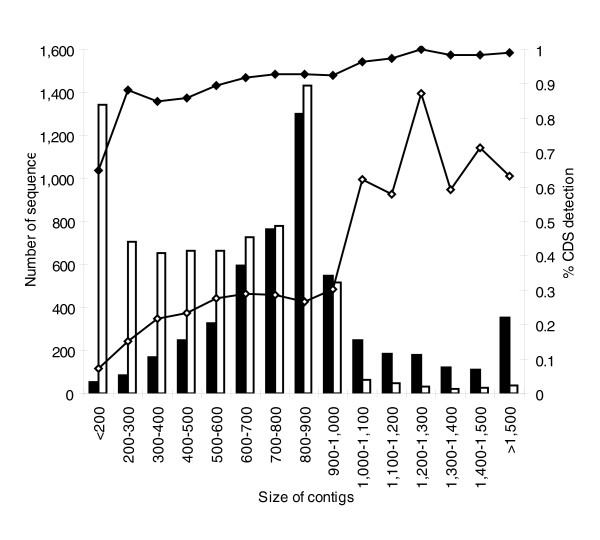
Size distribution of the 12,082 EST-derived unique transcripts from *A. pisum*. Contigs and singletons with (filled bars) or without (open bars) a significant hit have been selected with a cutoff value 10^-5 ^after a BLASTX on Uniprot. Size classes (in base pairs) were binned (for sequences less than 200 bp and more than 1,500 bp) to contain a minimum of 20 sequences for both 'hits' and 'no-hits' contigs. The curves (hits, filled diamonds; no-hits, open diamonds) show the percentage of contigs for which a coding sequence was predicted by FrameD. Contigs with no predicted coding sequences are presumably entirely UTR.

Putative functions corresponding to this pea aphid gene collection were reported by comparing these ESTs with the Uniprot database using BLASTX. Among the 12,082 unique transcripts 7,146 showed no homology with any other protein sequences (resulting in 59% of orphan sequences). This high representation of orphan genes might reflect the limited sequence quality delivered by single-pass sequencing (for example, too short sequences, wrong base calling leading to frameshift errors, and so on) [11]. Figure 2 indicates that pea aphid unique transcripts corresponding to orphan sequences were biased toward smaller sizes. Indeed, 25% of the orphan sequences and 2.5% of the sequences with a significant hit were less than 300 bp long, while 3% of the orphan sequences and 21% of the sequences with a significant hit were more than 1,000 bp long. Moreover, the median size for sequences with significant database hits was 838, whereas it was 596 for sequences without significant hits. Short sequence length cannot, however, explain our inability to detect homology for all no-hits sequences and some of these would actually contain coding genes that would be unique to aphids.

The 25 most abundant unique transcripts are listed (see Additional Data File 1 for the original data used to perform this analysis). Many correspond to housekeeping proteins (for example, ribosomal proteins and structural proteins) but some are orphan genes or represent more specific functions like the gene *takeout *(see Discussion). Among the 4,936 annotated unique transcripts, 4,080 and 3,977 had a significantly similar hit in *D. melanogaster *and *Anopheles gambiae*, respectively. Thus, less than 34% of the pea aphid unique transcripts have similarities to the model dipteran species *D. melanogaster*. Among these, 751 *D. melanogaster *genes (defined as having a FlyBase ID) correspond to more than one *A. pisum *contig. This suggests the occurence of several paralogs of many pea aphid transcripts.

Pea aphid unique transcripts were also annotated through the Gene Ontology (GO) classification [12] (Table 3). The GoToolBox statistical test was used to compare the distribution of the GO terms in pea aphid unique transcripts with the *D. melanogaster *homologs for the different GO terms. General processes ('Physiological' or 'Cellular Processes', as well as 'Cell Components' or 'Transporter Activity') are more highly represented in the aphid collection than in the fly. This is due to the high proportion of 'Binding' and 'Catalytic Activity' terms in the aphid collection. The depletion of 'Development' GO terms in the pea aphid collection was unexpected, as in the parthenogenetic females that we sampled, embryos develop continuously in the ovarioles [13]. We also found an over-representation of transcripts with 'Translational Regulator Activity' and an under-representation of transcripts with 'Signal Transducer Activity'. There is an absence of *A. pisum *unique transcripts from the 'Defense and Immunity' category: this may reflect the fact that the aphids were not challenged with pathogens or parasites. Several enzymes involved in degradation of bacterial cell wall have been detected, however.

### Separation of coding and noncoding sequences

Detection of coding sequences by a program (FrameD) based on hidden Markov models (HMMs) (also using similarity information for sequences that had hits in databases) allowed us to predict open reading frames (ORF) among the different categories of sequences (those with or without a hit). As expected, there was a high rate of ORF prediction in the former category (more than 96% for contigs of at least 1,000 bp, see Figure 2). There was, however, a small proportion of sequences with a hit (and yet probably containing an ORF) but without any coding sequence (CDS) predicted. The frequency of such false negatives slightly exceeded 10% for contigs less than 1,000 bp and peaked for the shortest ones. Failure to detect a CDS is probably linked with too short size of the coding region in these sequences (which are probably mostly untranslated region (UTR)), and is also possibly a result of a low EST coverage (short contigs are made of fewer ESTs). For sequences without any hit in the Uniprot database, the program also generated some CDS but at a markedly lower frequency. The frequency of detected CDS appeared to plateau at about 30% for short contigs (less than 1,000 bp) and then rose sharply at about 60% for longer sequences (see Figure 2). Probably, most of the short contigs without hits and without detected CDS are entirely made of untranslated region (UTR), while 'long' contigs with the same characteristics are either particularly long UTRs, or could be untranslated RNAs with a functional activity. Overall, we could therefore extract a large collection of coding sequences and 5' UTR and 3' UTR sequences, and analyze their compositional properties.

### GC content of different regions and microsatellites

The global mean GC content was 33% (SD = 9.3% for the 12,082 unique transcripts), indicative of an AT-rich genome. Extraction of CDS and their separation from 5' UTRs and 3' UTRs yielded estimates of nucleotide composition at the different codon positions and in noncoding parts of the contigs for 5,309 aphid unique transcripts. For comparative purposes we analyzed a subset of the *D. melanogaster *transcript sequences corresponding to putative homlologs to pea aphid contigs, which amounted to 3,443 different CDS in the fly. Within the CDS, we found a sharp difference in GC content between the two insect species, particularly at the synonymous third codon positions (34% and 69% GC for *A. pisum *and *D. melanogaster *respectively) (Table 4). The net difference between the two species (defined as %GC from *D. melanogaster *minus %GC from *A. pisum*) was 9.0%, 2.8%, and 34.4% at the first, second, and third synonymous positions, respectively. The small difference at the second codon positions is consistent with these sites typically being the most conserved (because a change at the second position is always nonsynonymous).

In contrast, a major compositional change between aphid and fly was observed at the third synonymous codon positions, which are typically more susceptible to evolutionary change. The relatively high dispersion of %GC3 (the percentage of G or C at the third codon position), as measured by a larger standard deviation in aphid sequences (see Table 4), leads us to expect a rather strong heterogeneity in base composition and codon usage. This will be the subject of a future paper. Finally, the estimated percentage of GC in the 5' UTRs of the transcripts (34.9%) is almost equal to that of the third codon position, and that of 3' UTRs is even lower (23.1%). Thus, overall, the pea aphid transcripts show a significant compositional shift from *D. melanogaster *in being more AT rich while *D. melanogaster *shows high GC richness at the third codon position [14,15].

A list of 921 perfect microsatellite motifs is presented (see Additonal data file 2 for the original data used to perform this analysis) with their location in 796 different unique transcripts. A large proportion of microsatellite loci were dinucleotide (453) and trinucleotide (442) whereas 26 tetranucleotide repeats were found in the database. This differs from the general pattern of dominance of dinucleotide repeats and rarity of trinucleotide repeats [16]. (AT)_n _repeats dominate in pea aphid ESTs. Information from our gene prediction analysis shows that 92.5% of these motifs are expected to locate in noncoding sequences (either in contigs with no gene detected, or in the 5' UTR or 3' UTR of a contig with a gene dectected). These observations are statistically consistent with a high AT richness of the pea aphid genome and the locations of most motifs in noncoding sequences that are even more AT rich. These microsatellites provide a large collection of potential markers for genetic mapping and analysis of quantitative trait loci.

### *In silico *gene expression analyses

We carried out *in silico *gene-expression profiling for each tissue used for cDNA library construction (see 3 for the original data used to perform this analysis). This statistical test was performed on the organ-specific cDNA libraries, with the exception of the Whole body - Multi stage library. A group of 135 unique transcripts was selected above the *R *threshold of 10, corresponding to a 1% error risk, based on a Monte-Carlo computation. We found that bacteriocytes and parthenogenetic embryos were rich in tissue-specific unique transcripts (58 and 52, respectively). Thus, while these two libraries showed the highest level of redundancy (see Table 2), they also contained many tissue-specific genes. Bacteriocyte-specific unique transcripts corresponded mainly to amino-acid metabolism and transport as well as defense reactions, and have been described in detail elsewhere [17]. A majority of the genes specifically expressed in the bacteriocytes - as judged by quantitative reverse transcription PCR (qRT-PCR) performed in [17] - were among the list of the unique transcripts selected *in silico *(for example, lysozyme, phenylalanine monooxygenase, and inorganic phosphate transporter).

The parthenogenetic embryos displayed a high redundancy of unique transcripts and, most surprisingly, 75% of these unique transcripts did not share a homolog with *D. melanogaster*. Some of these highly expressed transcripts do not share similarity with any other sequences in GenBank. This might indicate specialized differentiation processes specific to parthenogenetic embryogenesis in aphids [13]. Two transcripts encoding proteins homologous to *D. melanogaster *proteins involved in regulation of sex determination (Transformer-2) and dosage compensation (MSL3) were detected as specifically expressed in parthenogenetic early embryos. But, whether sex determination and dosage compensation mechanisms in aphids are similar to those of *Drosophila *or not remains unknown.

Transcripts specifically expressed in heads and antennae of the pea aphid were also identified. Among the head- and antennae-specific transcripts were several endocuticular proteins, which may suggest a special cuticle composition of the head and antennae, and/or the high cuticle ratio represented in these body parts compared with other analyzed organs.

## Discussion

Hemiptera (for example, true bugs, whiteflies, cicadas, and aphids) are characterized by modified piercing and sucking mouthparts that are used to suck plant juices or to bite animals, and include many agricultural and horticultural pests as well as biting and blood-sucking pests (for example, *Rhodnius prolixus*, the vector of Chagas' disease). The pea aphid EST collection described in this paper represents half of all the Hemiptera sequences deposited in GenBank (as of October 2005) and is an invaluable source of molecular markers (microsatellites) and protein-coding genes. The large collection of unique transcripts derived from these ESTs may represent a high percentage of the expected approximately 15,000 genes from the genome of *A. pisum*, as estimated from the gene content of other insect species. Hemiptera and Diptera have diverged for more than 300 million years [18] and only about one-third of the pea aphid unique transcripts have putative homologs with the two dipteran species *D. melanogaster *and *A. gambiae*. The pea aphid gene sequences show a marked AT enrichment at degenerate positions compared with those of Diptera. A detailed study elsewhere will analyze more precisely the patterns of codon usage and codon preferences in the aphid genome, to determine whether we find signs of adaptation of codon bias, as has been found in *D. melanogaster *[19]. *Buchnera *species, the aphids' primary endosymbiont, also exhibits a strong bias towards AT [20], contrary to the situation in most free-living enterobacteriaceae such as *Escherichia coli*.

The pest status attributed to many aphid species is largely the result of their biology, such as their particular mode of reproduction through cyclical parthenogenesis, their dispersal capacity through the induction of winged morphs, their transmission of viral and other plant diseases, and their rapid adaptation to insecticides and resistant host plants. Our large-scale EST project involving both the whole insect and specific tissues or organs is a first step in describing the cellular functions involved in these biological processes. The sequences derived from the whole-insect library provide an overview of the main cellular functions active in pea aphid parthenogenetic females. In contrast, the five other cDNA libraries from isolated organs focused on more specific functions in the head, antennae, digestive tract, bacteriocytes, and parthenogenetic embryos. This diversity of tissue types, as well as the use of non-normalization procedures, allowed digital analysis of gene expression. The *in silico *analysis of gene-expression patterns identified 135 genes putatively expressed in tissue-specific patterns. For example, our analysis revealed that the parthenogenetic embryos express a large number of orphan genes that are expressed at low levels or not at all in other tissues. The parthenogenic aphids of embryos represent a highly modified form of embryogenesis [13]. It is possible that these novel genes play specific roles in embryonic development and, if so, then this would conflict with the widespread view that embryonic development in insects reflects the action of conserved genes in distantly related species [21].

A second surprising result of our survey is the observation that transcripts for genes related to the *takeout *gene of *D. melanogaster *are expressed at high levels in pea aphids. The transcripts we found seem, in fact, to represent two paralogs that originated after the divergence of the common ancestor of aphids from the common ancestor of flies (data not shown). Both paralogs are found in both our collection of ESTs from *A. pisum *and the ESTs from another aphid, *Toxoptera citricida *[22]. *Takeout *shares amino-acid sequence similarity with a large class of proteins related to juvenile-hormone-binding proteins. *Takeout *is regulated both by circadian rhythms [23] and by the sex-determination pathway [24]. The gene is also induced under starvation conditions and prolongs survival under starvation conditions. In addition, a related protein, Moling, discovered in the tobacco hawkmoth *Manduca sexta *(also known as the tomato hornworm, hornblower or Carolina sphinx) [25], is regulated by juvenile hormone, ecdysone, and starvation. Given the very high levels of expression of these ESTs in our collections, it is likely that these *takeout*-related genes have an unexpected and important role in aphid biology.

*A. pisum *belongs to the tribe Macrosiphini of the family Aphididae. Some of the most important aphid agricultural pests, such as the Russian wheat aphid *Diuraphis noxia *and the peach-potato aphid *Myzus persicae*, belong to the Macrosiphini. There are more limited EST collections from two species of the tribe Aphidini, also of the family Aphididae, *Rhopalosiphum padi *and *Toxoptera citricida*, and many of these ESTs share homologs with ESTs in our collection. This indicates that our *A. pisum *unique transcript set is a valuable genomic resource that will inform studies of many pest species in the entire family Aphididae, which radiated 30-80 million years ago [26].

## Conclusion

This work demonstrates the importance of characterizing transcript collections (codon usage, putative paralogs, orphan sequences) of an agronomical important pest that diverged a long time ago from model organisms. To pursue this effort, the aphid EST collection is an important resource for the future annotation of the pea aphid genome, which was selected in 2005 by the National Human Genome Research Institutefor sequencing.

## Materials and methods

### Nomenclature

Several cDNA libraries were constructed from different *A. pisum *genotypes and different biological sources. The names and descriptions of the different cDNA libraries are listed in Table 1. Bacteriocyte ESTs have already been described elsewhere [17]. For some libraries, different codes were used to discriminate between the different sequencing centers.

### Biological material

The pea aphid lines YR2 [27] and P123 (A. Frantz, M. Plantegenest, and J.C.S. unpublished work) were reared on *Vicia fabae *in the laboratory. They were maintained in conditions of continuous parthenogenetic reproduction under long photoperiod (16 hours light/8 hours dark) and warm temperature (18°C). For libraries ApAL3SD and ApHL3SD, insects were placed in sex-inducing conditions using a standard protocol at short photoperiod [27] in order to enrich the cDNA libraries in transcripts expressed during induction of sexual reproduction. The strain ISO was described in [17]. The clone LL01 of *A. pisum *used for 15 years for all the Lyon group's physiological and toxicological experiments, was collected in 1987 near Lusignan (France) from an alfalfa field, and has been continuously reared since then on broad bean seedlings in parthenogenetic conditions. An anonymous clone collected in Cambridge, UK, that has since been lost, was used to generate the cDNA libraries ApMS, 14419 and 14436.

### cDNA libraries

The multistage cDNA library (ApMS; 14419; 14436) was constructed from poly(A)^+ ^RNA extracted from the whole bodies of a mixture of wingless and winged parthenogenetic individuals at the five different developmental stages (first through fourth instar larvae and adult). All other cDNA libraries were prepared from total RNA. Transcripts from antennae and heads were extracted from third instar larvae of parthenogenetic individuals (50 individuals for heads and 200 individuals for antennae) reared under either long (ApHL3LD, ID0ACC and ID0AEE) or short (ApAL3SD, ApHL3SD) photoperiod. Dissection of antennae and heads (without antennae) was performed under a dissecting microscope on frozen insects. Parthenogenetic embryos (*n *= 150) were dissected from adult parthenogenetic females under a dissecting microscope: only the three to four earliest stages of embryos were collected. The digestive tract cDNA library was constructed from the guts of young parthenogenetic adult females. For dissection of guts, insects were immobilized in batches of 10 or 20, and guts were carefully dissected in ice-cold PBS, pulled out through the head after liberating the hindgut by cutting the anal part. Freshly dissected gut batches (*n *= 50) were deep-frozen by plunging the plastic Eppendorf tube in liquid nitrogen, and stored at -80°C until extraction (*n *= 1,200).

Total and poly(A)^+ ^RNAs were extracted either by the guanidinium-salt-phenol chloroform procedure as described [22] or by using the RNeasy Plant Mini Kit (Qiagen, Hilden, Germany) in the RTL extraction buffer. Plasmid cDNA libraries were constructed with the Creator Smart cDNA Library Construction Kit (BD Biosciences Clontech, Palo Alto, USA). Lambda Uni-ZAP libraries and mass excision of plasmids were obtained as described [22]. The bacterial glycerol stocks are archived at the Horticultural Research Laboratory (USA), the INRA-Rennes Laboratory (France), the INRA-Lyon Laboratory (France), and RIKEN (Japan).

### Sequencing, sequence processing and annotation

Sequencing reactions were performed either on purified plasmids [22,28] or on PCR-amplified products [29] using the ABI PRISM BigDye technology (Applied Biosystems, Foster City, USA). Sequences were analyzed on different types of automated multicapillary sequencers (ABI 3100, ABI 3700 and ABI 3730xl) in the different sequencing centers (see Table 1). ESTs were deposited in GenBank (dbEST) or the Data Bank of Japan (DDBJ) (see Table 2).

Each EST was then analyzed through a pipeline for cleaning and clustering, as described in [30]. Phred was used from traces to predict sequences. Adaptor and vector were localized using cross_match, an implementation of a Smith-Waterman algorithm using default matrix (1 for a match, -2 penalty for a mismatch), with mean scores of 6 and 10 respectively. Sequences were then trimmed following three criteria: vector and adaptor, poly(A) tail or low quality (defined as at least 15 among 20 bp with a phred score below 12) [30]. Identification of contaminant sequences was also performed by cross_match, using the default matrix (1 for a match, -2 penalty for a mismatch). *E. coli *and yeast sequences were eliminated with a score greater than 100 against complete corresponding genomes retrieved from Genbank. Ribosomal sequences were eliminated by comparison to an invertebrate ribosomal sequence library (extracted from GenBank) using a cross_match score of 50. Finally, as aphids live in symbiosis with *Buchnera *bacteria [31], *A. pisum *ESTs were compared to the *Buchnera *species APS genome [32] and those sequences presenting a match score of greater than 50 were eliminated.

Of the remaining sequences, clustering was performed using Biofacet [33], based on the criteria of 96% similarity in 80 bp. Clusters were then aligned in contigs and consensus sequences were obtained using Cap3 [34]. The contigs were analyzed through a NCBI-BLASTX [35] against Uniprot [36]: only significant matches with a Phred 20 score and with an *E*-value of less than 1.0e^-5 ^were considered for report. All data were stored in a freely accessible relational database [10]. A tutorial is available [37] and all sequences (contigs and singletons) can be downloaded [38].

Description of the contig collection was facilitated by a link to the GO database through the Amigo browser [12] to search for contigs belonging to different GO categories. Furthermore, GO categories were also used to search for over- or under-represented GO terms in the pea aphid contig dataset using GOToolBox [39]. This program statistically associates over- or under-represented GO terms with a given contig, compared to the distribution of these terms among the annotation of the complete genome. For this analysis, as the *A. pisum *genome is not yet available, *D. melanogaster *was selected as the reference genome. Thus, only the *A. pisum *contigs having a hit with a *D. melanogaster *gene have been computed. Putative orthologous peptides of the pea aphid unique transcript sequences were extracted from FlyBase using BLASTX (greater than an E-value threshold of 1e-5) and used for the comparison between the *D. melanogaster *and *A. pisum *distribution of GO terms.

### EST frequencies

A statistical analysis of the frequency of each EST in the different cDNA libraries was performed to compare gene-expression levels in different tissues. Groups of contigs and singletons were first done on the basis of their EST composition (frequency per cDNA library), using the K-means algorithm through a boostrap aggregating function provided by the R package. Whole contig groups or selected K means were then statistically analyzed to identify putative differentially expressed genes [40]. The null hypothesis states that the frequency of a gene transcript is the same in each library, the variation in numbers of ESTs from different libraries in a given contig being due to sampling error. The significance of the R-value test is determined either by a large deviation rate or a Monte Carlo simulation. Any new putative differentially expressed genes can be identified on a web interface (ADEL -Analysis of the Distribution of ESTs in cDNA Libraries) developed at Genoplante-Info [41].

### Separation of coding and noncoding sequences and GC content

Identification of ORFs among the collection of contigs was intended to be by analysis separately of the compositional properties of different sections of the DNA sequences - CDS or UTR. As a first step, we used similarity information from a BLASTX pairwise comparison between the collection of contigs and the *D. melanogaster *coding genome. High-scoring pairings indicate that the coding frame in the contig had a hit in *D. melanogaster*, and this is followed by a search for the first 5' methionine and the first 3' stop codon. Because such reconstruction was limited to contigs with a hit in *D. melanogaster*, however, and was error prone (for example, no fine detection of potential frameshifts and poor identification of the starts and ends of genes), we used a randomly chosen sample of 270 putative complete CDS constructed in the first step (genes starting with a methionine and ended by a stop codon) to train a program based on HMMs. This program, FrameD, has been specially designed to recognize genes among genomes of biased composition and among noisy data (such as ESTs), predicting frameshifts and proposing corrections in such cases [42]. After training, the program was run on the complete set of contigs, using both the information from the matrix of the training set and similarity information (BLASTX hits if any). The program can be used online at [43], setting the probabilistic model to 'Apisum'.

### Simple-sequence repeats and SNPs

Perfect simple-sequence repeats have been extracted from the pea aphid unique transcripts by application of an exact pattern algorithm. Only motifs with at least seven repetitions of two nucleotides, six repetitions of three nucleotides or five repetitions of four nucleotides were retrieved.

## Additional data files

The following additional data are available online with this paper. Additional Data File 1 contains the list of the 25 most abundant *A. pisum *unique transcripts from the 12,082 collection. Additional Data File 2 contains the list of the 921 perfect microsatellite motifs found in the 12,082 *A. pisum *contigs. Additional Data File 3 contains the description by cDNA libraries of the 135 contigs specific to a given *A. pisum *tissue.

## Supplementary Material

Additional data File 1 A list of the 25 most abundant *A. pisum *unique transcripts from the 12,082 collection. Click here for file

Additional data File 2 A list of the 921 perfect microsatellite motifs found in the 12,082 *A. pisum *contigs Click here for file

Additional data File 3 A table giving the description by cDNA libraries of the 135 contigs specific to a given *A. pisum *tissue Click here for file

## Figures and Tables

**Table 1:** List of pea aphid libraries used for the EST database

Biological source	Aphid line	Library	RNA	Vector	Sequencing center	Accession Number
Antennae	YR2	ApAL3SD	Total	pDNR-LIB	Roscoff	[GenBank:CN748946 to CN749908]
	YR2	ID0AEE	Total	λ Uni-Zap	Genoscope	[GenBank:CV844624 to CV850040]
Bacteriocyte	ISO	ApBac	Total	λ FLC-I	RIKEN	[DDBJ:BP535536 to BP537955]
Digestive tract	LL01	ApDT	Total	pDNR-LIB	Roscoff	[GenBank:CN749909 to CN751017]
Head	YR2	ApHL3LD	Total	pDNR-LIB	Roscoff	[GenBank:CN752448 to CN753369]
	YR2	ApHL3SD	Total	pDNR-LIB	Valencia	[GenBank:CN751018 to CN752447]
	P123	ID0ACC	Total	λ Uni-Zap	Genoscope	[GenBank:CV828453 to CV839072]
Parthenogenetic embryo	YR2	ID0ADD	Total	λ Uni-Zap	Genoscope	[GenBank:CV839157 to CV844599]
Whole-body, multistage	Unknown	ApMS; 14419; 14436	Polya+	λ Uni-Zap	Genoscope and Fort Pierce	[GenBank:CN753369 to CN764460, CF546452 to CF546552, CF587442 to CF588411, CN582088 to CN587684]

**Table 2:** Number of raw sequences, selected ESTs, sizes, contigs formed, and redundancy in *A. pisum *EST database

Biological source	Library	EST	Rejected				Selected	M bp	Contig	Singletons	Redundancy
								
			Bacterial	rRNA	Short sequences	Vector sequences					
Antennae	ApAL3SD	1,031	10	39	84	0	898	398	305	283	34.52
	ID0AEE	5,424	23	431	46	1	4,923	622	1,037	2,414	29.90
Bacteriocyte	ApBac	2,345	1	0	3	0	2,341	871	275	40	86.54
Digestive tract	ApDT	1,184	52	333	94	0	705	403	267	211	32.20
	ApHL3LD	1,245	24	30	359	0	832	394	366	201	31.85
Head	ApHL3SD	2,068	7	33	739	0	1,289	363	382	438	36.38
	ID0ACC	10,706	3	902	221	3	9,577	574	2,012	1,564	62.66
Parthenogenetic embryo	ID0ADD	5,473	136	541	105	0	4,691	717	210	151	92.30
Whole body, multistage	ApMS; 14419; 14436	17,964	479	1455	382	0	15,648	716	5153	3027	47.72
GenBank	mRNA	3	0	0	0	0	3	1220	2	1	0.00
Total		47,443	735	3,764	2,033	4	40,907	628	4,300	7,782	70.46

M bp: mean size of ESTs in base pairs.

**Table 3:** Gene Ontology annotation of pea aphid unique transcripts after GoToolBox statistical analysis

Gene Ontology	Putative orthologs set	*Drosophila *genome	Corrected *p* value
**Biological process**	2,397	10,032	
Physiological process	2,260	7,986	En (2e^-112^)
Cellular process	2,201	7,727	En (9e^-101^)
Regulation of biological process	431	1,583	En (8e^-4^)
Growth	35	98	En (4e^-5^)
Pigmentation	19	51	/
Behavior	129	637	/
Reproduction	162	812	/
Development	470	2,227	D (4e^-4^)
Unknown	49	833	D (2e^-46^)
**Cellular component**	1,684	7,428	
Cell	1,532	5,150	En (3e^-124^)
Protein complex	733	1,747	En (9e^-98^)
Organelle	1,020	2,966	En (3e^-84^)
Extracellular matrix	23	82	/
Extracellur region	85	450	/
Unknown	73	1,865	D (3e^-140^)
**Molecular function**	2,397	10,104	
Catalytic activity	1,290	4,070	En (1e^-52^)
Binding	1,334	4,301	En (8e^-49^)
Structural molecule activity	298	757	En (8e^-23^)
Translation regulator activity	54	92	En (5e^-12^)
Transporter activity	372	1,237	En (9e^-8^)
Enzyme regulator activity	110	379	/
Antioxydant activity	16	39	/
Motor activity	26	88	/
Transcription regulator activity	187	841	/
Signal transducer activity	215	1,093	D (1e^-3^)
Unknown	63	1,798	D (1e^-144^)

The set of *A. pisum *contigs orthologous to *D. melanogaster *sequences have been compared to the whole set of *D. melanogaster *genes using FlyBase Gene ontology terms. The last column indicates the *p* value of the hypergeometric test. En, enhanced and D, depleted in *A. pisum *transcripts. /, no bias.

**Table 4:** Base composition (%GC) at different positions for reconstructed coding sequences of the collection of aphid contigs and their putative homologs (best hits) in *D. melanogaster*

		%GC1	%GC2	%GC3s	5' UTR	3' UTR
*A. pisum*	Mean	47.4%	37.0%	34.5%	34.9%	23.1%
	SD	6.6%	7.2%	14.2%	10.0%	8.3%
*D. melanogaster*	Mean	56.4%	39.8%	68.8%	ND	ND
	SD	4.6%	5.7%	9.0%	ND	ND
